# Genetic Contributions to Intergroup Responses: A Cautionary Perspective

**DOI:** 10.3389/fnhum.2012.00223

**Published:** 2012-08-06

**Authors:** Kyle G. Ratner, Jennifer T. Kubota

**Affiliations:** ^1^Department of Psychology, New York UniversityNew York, NY, USA

We live in an era where genomic information can be collected with the ease of a saliva sample and the cost of genotyping is plummeting (Hirschhorn and Daly, 2005; Quinque et al., 2006). Intergroup researchers interested in incorporating biological approaches into their methodological toolbox are thus faced with the question of whether molecular genetics can provide novel insight into their understanding of people's responses to members of other groups. In the current commentary, we stitch together human and animal neuroscience with insight from molecular biology to posit mechanisms through which genetic variation and life experience may give rise to responses during intergroup situations. We then discuss avenues for empirical investigation and urge for responsible research practices that take into consideration the negative societal consequences that can result from overinterpreting genetic data.

Intergroup phenomena, such as discrimination and ethnic violence, emerge at the interindividual level, and as a result, cannot be fully explained by intrapsychic processes. However, in order to understand the effects of intergroup influences on the individual, it is useful to examine the processes unfolding in the person's mind and brain. For this reason, researchers have increasingly combined experimental social psychology and neuroimaging techniques to dissect the neural basis of affective and cognitive mechanisms that contribute to intergroup responses. This approach has revealed many interconnected, but dissociable, brain regions involved in perceiving, evaluating, and regulating behaviors toward other people, including the amygdala, fusiform gyrus, anterior cingulate cortex, and various parts of the prefrontal cortex. For comprehensive reviews of the existing neuroscience research pertaining to intergroup relations we direct readers to Amodio and Ratner (2011), Cunningham and Van Bavel (2009), Ito and Bartholow (2009), and Kubota et al. (2012).

To understand how these neuroimaging findings might relate to genetics, it is important to recognize that communication between neurons is facilitated by neurochemicals, such as neurotransmitters (e.g., serotonin, dopamine), neurotrophic factors (e.g., BDNF), and hormones (e.g., cortisol, testosterone, and oxytocin). The enzymes that synthesize these molecules, the receptors to which they bind, and the reuptake mechanisms and enzymes that determine their availability are all proteins that are coded for by genes (Way and Gurbaxani, 2008). Thus, the cellular and molecular levels of analysis, although not currently considered by most intergroup researchers, have the potential to provide unique insight into how genetic variation might influence intergroup responses.

Genes are biologically meaningful segments of DNA (Snyder and Gerstein, 2003). Each gene consists of a sequence of nucleotides. Frequent variations in the ordering, number, type, and repetition of nucleotides are called polymorphisms. A single gene can have many different types of polymorphisms (den Dunnen and Antonarakis, 2001; Gibson and Muse, 2002). In order for polymorphisms to influence cellular functioning, their genetic code has to be transcribed into RNA and this RNA needs to be translated into amino acids. The type of amino acids that are produced and their configuration determine the form of the resultant proteins (e.g., enzymes, receptors; Crick, 1958).

Gene expression occurs when biochemical processes within a cell stimulate transcription factors that bind to particular DNA motifs (i.e., specified nucleotide sequences) in the promoter region of a gene. Extracellular events can control genomic responses through receptor-mediated channels (Cole, 2009). Relevant to the present concerns, social stressors, such as interacting with unknown outgroup members, have been shown to elevate levels of the hormone cortisol (Page-Gould et al., 2008; Amodio, 2009). Other research indicates that cortisol binds to corticosteroid receptors and then the bound receptors translocate to the nucleus and act as transcription factors. One effect is that they bind to sites in the promoter of the serotonin transporter gene to trigger the synthesis of proteins that control the reuptake of extracellular serotonin. The availability of serotonin has important effects on an individual's emotional responses and also indirectly contributes to serotonin regulation by influencing hypothalamic–pituitary–adrenal (HPA) pathways that release cortisol (Glatz et al., 2003; Heisler et al., 2007; Way and Gurbaxani, 2008). Thus, at the level of gene expression there is a bidirectional relationship between the genetic code and the environment.

The expression of a gene can also be influenced by epigenetic factors, such as DNA methylation and histone modification (Goldberg et al., 2007). DNA methylation occurs when a methyl group attaches to the promoter region and blocks DNA transcription. Environmental factors (e.g., stress) can increase DNA methylation, and thus have long-term effects on gene expression. Research in rodents has shown that DNA methylation can persist through the reproductive process, and as a result, can influence gene expression across generations (Meaney and Szyf, 2005; Champagne, 2008). For instance, rat pups raised by stressed mothers demonstrate increased methylation of the gene for BDNF and this methylation is passed on to offspring (Roth et al., 2009). BDNF is a nerve growth factor that increases synaptic plasticity throughout the nervous systems and has been shown to influence amygdala and prefrontal cortex responses to learned categories (Soliman et al., 2010; Forbes et al., 2011).

By tracing pathways in the brain that link the social context to genetically mediated cellular processes, it becomes clear that even the most basic intergroup responses (e.g., a negative feeling) reflect a vast amount of inputs and there is a bidirectional relationship between genes and the environment. Thus, there is no one-to-one gene to phenotype relationship that should be expected. This logic is matched by the reality that single genes often account for a small amount of variability in complex psychological phenomena (Risch and Merikangas, 1996; Colhoun et al., 2003; Hattersley and McCarthy, 2005).

It is possible that networks of gene variants could be inherited together, due to evolutionary pressures, and these genes collectively nudge people toward life experiences and attitudes that result in a particular aversion to outgroup members or a strong sense of ingroup loyalty. This possibility would be consistent with evidence supportive of gene-culture coevolution effects on emotional reactivity and social interdependence (Chiao and Blizinsky, 2010; Kim et al., 2010; Way and Lieberman, 2010). However, even if it is discovered that inheritance of multiple genetic polymorphisms increases the likelihood that a person will exhibit a certain intergroup response, any deterministic claims should be met with a high level of scrutiny. There are many steps through which countervailing influences could take hold and the polygenetic basis of diseases and traits with a recognized heritable component (e.g., cardiovascular disease and height) have proven difficult to establish (Ioannidis, 2009; Lango Allen et al., 2010). The prominence of the eugenics movement during parts of the nineteenth and twentieth centuries stands as a reminder of the perils of essentializing genetic differences (Duckitt, 1992; Eberhardt, 2005).

Although we believe that a deep integration of advances in molecular biology into social neuroscience research will ultimately show that genes are not sufficient to produce intergroup reactions, we hope that such efforts usher in a rival of Lewinian psychology (Lewin, 1936, 1943). Group psychologists have long treated person and situational variables independently (Duckitt, 1992), mainly because from a psychological level of analysis it is difficult to understand how they fit together. However, neuroscience can serve as a lingua franca connecting the social and genetic levels of analysis. At the level of molecules and cells, genetic and psychological factors can be considered in the same conceptual space and their interplay is illuminated.

A natural starting point for testing empirical predictions about the relationship between genes and intergroup reactions is through a candidate gene approach (Moffitt et al., 2005). Such an approach uses findings from animal research and pharmacological manipulations to theoretically predict which genes should be involved in a psychological process. In the only published study to examine genetic factors related to intergroup responses, Forbes et al. (2011) measured gender stereotypes in genotyped frontal lobe patients. They found that a BDNF polymorphism that enhances synaptic plasticity was associated with better regulation of implicit gender stereotypes in patients with relatively small OFC lesions. The same polymorphism related to less explicit stereotyping in participants with relatively small DLPFC lesions. Given reported associations between amygdala-mediated affective arousal and polymorphisms in several genes, including serotonin transporter, COMT, and BDNF genes (Hariri et al., 2005; Drabant et al., 2006; Soliman et al., 2010; Hartley et al., 2012), there is reason to believe that a candidate gene approach might also identify genetic contributions to emotionally charged group-based responses, such as anxious arousal during intergroup interactions, stereotype threat, ingroup favoritism, and outgroup derogation.

Due to the likelihood that most complex processes involve a multitude of genetic influences and our ability to identify them *a priori* is limited by the complexity, an alternative data-driven approach called genome-wide associations has gained in popularity (Hirschhorn and Daly, 2005; Pearson and Manolio, 2008). This method involves correlating a psychological factor with variation across the entire genome. Despite the allure of association studies, both candidate gene and genome-wide findings have proven difficult to replicate (Hattersley and McCarthy, 2005; Shriner et al., 2007; Williams et al., 2007).

One possible reason for replication failures is that epigenetics have largely not been taken into account. Technological advances that are enabling the characterization of gene expression (transcriptomics) and downstream production of proteins (proteomics) have given rise to a burgeoning area of research that has the potential to facilitate understanding of genetic pathways that are functionally related to psychological processes (Zivy and de Vienne, 2000; Figeys, 2002; Chaussabel et al., 2010). This work is complicated, however, by the fact that methylation patterns vary across the body and across different regions of the brain (Xin et al., 2010; Aberg and van den Oord, 2011).

The joint consideration of multiple genes, epigenetic information, and cultural and social psychological variables requires large samples to achieve sufficient statistical power. Data collection via the internet provides a natural solution for addressing this problem (Eriksson et al., 2010; Gibson and Copenhaver, 2010). Internet-based behavioral assays can reach thousands of research participants, are relatively easy to administer, and a growing number of cases have demonstrated data quality comparable to experiments carried out in controlled laboratory settings (Nosek et al., 2007; Buhrmester et al., 2011; Mason and Suri, 2012). Moreover, information from online social networks (e.g., facebook) could be used to objectively characterize important intergroup factors, such as people's contact with members of different groups and their family and friends’ attitudes and exposure to diversity (Wright et al., 1997; Pettigrew and Tropp, 2006; Saribay and Andersen, 2007; Turner et al., 2007). Investigators could also examine whether the genes of individuals in a person's network influence that person's gene expression and intergroup behavior (for a demonstration of a related concept outside of the intergroup context, see Fowler et al., 2011). Furthermore, it is only a matter of time before researchers collect location information and physiological data (e.g., saliva, pupil dilation, anxiety in speech patterns) through mobile devices, and dynamically assess intergroup processes in the real world. Eventually, efforts will not be impeded by sample size, cost, or the difficulty of acquiring data, but by the bioinformatic techniques capable of making sense of these data.

Although the true value of incorporating genetics into the scientific understanding of intergroup relations is currently unknown, technologies are beginning to converge and allow for an environment capable of testing questions of gene and environment interactions that could not be empirically considered even a decade ago. The theoretical possibilities are vast, but as psychologists explore these issues they should do so judiciously. Single candidate genes and even multiple gene interactions are distal predictors of behavior that account for small portions of variance. As a result, any indication that genetic polymorphisms explain some variance in a prejudicial response should not be sensationalized as evidence for “racism genes.” Inheriting certain genes does not necessarily indicate that an individual will behave in a prescribed fashion across all situations or even in a particular instance. The complexity of mapping the interplay of social factors and genes should give people pause before using this insight to predict everyday behavior. Future researchers should join with legal scholars and philosophers to fully explore the moral implications of such endeavors.
